# A randomised, controlled, crossover study of the effect of diet on angiopoietin-like protein 4 (ANGPTL4) through modification of the gut microbiome

**DOI:** 10.1017/jns.2016.38

**Published:** 2016-12-06

**Authors:** Trine Blædel, Jacob B. Holm, Ulrik K. Sundekilde, Mette S. Schmedes, Anne L. Hess, Janne K. Lorenzen, Karsten Kristiansen, Trine K. Dalsgaard, Arne Astrup, Lesli H. Larsen

**Affiliations:** 1 Department of Nutrition, Exercise and Sports, Faculty of Science, University of Copenhagen, Frederiksberg, Denmark; 2 Department of Biology, Faculty of Science, University of Copenhagen, Copenhagen, Denmark; 3 Department of Food Science, Faculty of Science and Technology, University of Aarhus, Kirstinebjergvej 10. 5792 Årslev/Blicher allé 20, 8830 Tjele, Denmark

**Keywords:** Angiopoietin-like protein 4, Lipid metabolism, Lipoprotein lipase, Obesity, Gut microbiome, %E, percentage energy, ANGPTL4, angiopoietin-like protein 4, HIF-1α, hypoxia-inducible factor 1α

## Abstract

Angiopoietin-like protein 4 (ANGPTL4) is a lipoprotein lipase inhibitor that is involved in lipid metabolism and angiogenesis. Animal studies have suggested that the ANGPTL4 protein is modulated by the gut microbiota, possibly through increased concentrations of SCFA, such as C4, found in whole-fat milk or as a result of fermentation of inulin. This study investigated whether a standardised diet either high in fat content or supplemented with inulin powder would increase plasma ANGPTL4 in overweight men and whether this increase was mediated through a compositional change of the gut microbiota. The study had a crossover design with three arms, where participants were given a standardised isoenergetic diet supplemented with inulin powder, whole-fat milk or water (control). Plasma and urine samples were collected before and after each intervention period. Faecal samples and adipose tissue biopsies were collected after each intervention period. The study included twenty-one participants of whom eighteen completed the study. The dietary interventions did not change ANGPTL4 plasma concentration, nor was plasma ANGPTL4 associated with plasma lipids, TAG or NEFA concentration. The relative abundance of bifidobacteria following the inulin diet was higher, compared with the control diet. However, the changes in microbiota were not associated with plasma ANGPTL4 and the overall composition of the microbiota did not change between the dietary periods. Although weight was maintained throughout the dietary periods, weight was negatively associated with plasma ANGPTL4 concentration. In the adipose tissue, *ANGPTL4* expression was correlated with leptin expression, but not with hypoxia-inducible factor 1α (*HIF-1α*) expression.

Dysregulation of TAG and fatty acid metabolism increases the risk of CVD and type 2 diabetes^(^^1^^)^ and stringent regulation of lipid metabolism is a prerequisite for maintaining metabolic health. Angiopoietin-like protein 4 (ANGPTL4) is a lipoprotein lipase inhibitor that regulates lipid metabolism and storage^(^^2^^)^ and as such ANGPTL4 is a potential target for interventions to modify the risk of developing obesity-associated diseases.

ANGPTL4 is expressed in several tissues in humans, including the liver, adipose tissue and intestine^(^^3^^)^. The protein is negatively associated with TAG concentration in animal models^(^^4^^,^^5^^)^. In humans, plasma ANGPTL4 is positively associated with concentrations of NEFA and increased lipolysis^(^^6^^,^^7^^)^, while carriers of an *ANGPTL4* loss-of-function mutation have lower TAG concentration than non-carriers^(^^8^^,^^9^^)^. Additionally, ANGPTL4 is a regulator of angiogenesis and mediator of the effect of hypoxia in the adipose tissue, which influences the lipid-storing capacity of adipose tissue^(^^2^^)^.

ANGPTL4 can be regulated by diet^(^^6^^,^^10^^,^^11^^)^. A short-term (3 d) human study observed an up-regulation of plasma ANGPTL4 in response to a high-fat diet (69 % energy (E%) as fat), a very low-energy diet (471 kcal/d; 1971 kJ/d) and fasting^(^^6^^)^, which suggests that an increased concentration of NEFA in the bloodstream up-regulates expression of *ANGPTL4*. This up-regulation has been shown *in vitro* as expression of *ANGPTL4* is up-regulated in response to exposure to fatty acids, including 16 : 0, 18 : 1*n*-9 and 20 : 4*n*-6 in cell studies^(^^12^^,^^13^^)^.

Regulating ANGPTL4 by diet-induced modifications of the gut microbiota offers the potential of indirectly modulating ANGPTL4 without intake of excessive amounts of fat or by extreme fasting. Conventionalisation of germ-free mice has shown down-regulation of intestinal *ANGPTL4* expression^(^^14^^)^ whereas *Lactobacillus paracasei* F19 has been shown to increase concentrations of circulating ANGPTL4 in mice^(^^10^^)^. The observed up-regulation is probably caused by an increase in the production of SCFA, particularly C4, which has been shown to increase *ANGPTL4* expression *in vitro*^(^^15^^)^. An *in vivo* up-regulation of C4 could be induced by increasing the relative proportion of C4-producing bacteria, such as the *Roseburia* species^(^^16^^)^, by supplying prebiotics in the diet^(^^17^^)^.

Collectively, the evidence suggests that ANGPTL4 is an important regulator of lipid metabolism, with potential consequences for health, and that dietary interventions may alter circulatory ANGPTL4. However, it is unclear whether moderate dietary interventions of intermediate duration in human subjects can modify ANGPTL4. Further, the role of the gut microbiota and adipose tissue in regulation of ANGPTL4 has not been examined in humans.

Thus, the objective of the study was to investigate whether a standardised diet either high in milk fat or supplemented with inulin powder would increase plasma ANGPTL4 in overweight men and whether this increase was mediated through a compositional change of the gut microbiome.

## Methods

### Study design

The study was a randomised, controlled, crossover study with three 21 d intervention periods. The intervention periods were separated by a wash-out period of at least 21 d. The participants completed an investigation day at the start and end of each intervention period as well as a brief visit 24 h after the start of each intervention period. All study visits were carried out at the Department for Nutrition, Exercise and Sports, Copenhagen University, Denmark. Recruitment commenced August 2013 and was completed in January 2014. The study was completed in July 2014.

This study was conducted according to the guidelines laid down in the Declaration of Helsinki and all procedures involving human participants were approved by the Regional Ethical Committee of Copenhagen (no. H-3-2013-086). Written informed consent was obtained from all subjects.

The study was registered at www.clinicaltrials.gov with the identifier NCT01913678 and the Danish Data Protection Agency (2007-54-0269).

### Subjects

The inclusion criteria required the participants to be men, healthy, aged 23–45 years, have a BMI of 25–35 kg/m^2^ and a body fat percentage >20 %. Exclusion criteria included the presence of chronic diseases, such as diabetes, CVD, irritable bowel syndrome, colitis ulcerosa, Crohn's disease and other chronic diseases likely to affect the outcomes of the study, as well as milk allergy or lactose intolerance, intolerance towards inulin, elevated fasting glucose (≥7·0 mmol/l or non-fasting glucose ≥11 mmol/l), the use of antibiotics 2 months before commencement of study, the use of dietary supplements including multivitamins during the entire study period, smoking, excessive physical activity (>10 h of strenuous physical activity per week), use of prescription medicine, which could affect the results of the present study including systemic glucocorticoids and the use of lipid-lowering agents or medication with contraindications for a high-fat diet, blood pressure >140/90 mmHg, blood donation <1 month before study commencement and during the study period, simultaneous participation in other clinical studies or the inability, physically or mentally, to comply with the procedures required by the study protocol as evaluated by the study staff.

Participants were recruited through local newspapers and flyers at educational institutions in the Copenhagen area. Informed consent was signed by all participants before the commencement of any study procedures. Subsequently, the participants completed a screening visit to assess biological and anthropometric inclusion and exclusion criteria. Weight, height, blood glucose, body fat percentage and blood pressure were measured at the screening. Potential participants that fulfilled the inclusion and exclusion criteria were invited to participate in the study.

### Interventions

The diets were isoenergetic and participants were on one of two energy levels (13 or 15 MJ/d) according to the BMR, which was calculated based on age, sex, height, weight using the Schofield equation for men >18 years^(^^18^^)^ and adjusted for self-reported physical activity level. The participants were instructed to weigh themselves weekly to ensure weight stability and additionally to register their intake of fluids, physical activity level and bowel movements. If weight changes above 0·5 kg/week occurred, the diet was adjusted to match the energy requirement of the individual.

The diets were composed of typical Danish dishes. The dietary items for each day were repeated in modules of 4 d, so that each module was repeated four or five times during the 21 d of intervention. Relative macronutrient composition was held constant between energy requirement levels. The diets were designed so that the high-inulin diet differed only from the control diet in the carbohydrate type, with 13–15 g of inulin fibre added to the high-inulin diet (high-inulin diet E%: carbohydrate: 55 %, fat: 35 %, protein: 15 %). The total carbohydrate energy percentage was identical between the two diets (control E%: carbohydrate: 55 %, fat: 35 %, protein: 15 %). The high-dairy diet differed from the control diet in fat energy percentage and type (high-dairy diet E%: carbohydrate: 45 %, fat: 40 %, protein: 15 %). Protein quantity and quality were similar in the three dietary periods and the source of fibre was identical between groups besides the inulin supplement in the high-inulin group.

The participants were provided with all dietary items including beverages twice weekly throughout the three intervention periods. They were instructed not to consume anything besides the dietary items provided except for habitual intake of non-energy beverages (water, coffee, tea and artificially sweetened drinks). During the wash-out periods, the participants were asked to maintain their habitual dietary intake and level of physical activity. Depending on the energy requirement of the participants, 13 or 15 g of flavourless and odourless Inulin HPX^®^, designated safe for consumption (BENEO-Orafti, Alsiano A/S) were dissolved in 500 ml of 90°C warm water until the powder had dissolved. Subsequently, the solution was cooled at 5°C overnight. Two drops of apple aroma were added to ensure that there was no difference in taste of the solutions. Supplemental water was treated identically to the inulin solution, without the addition of inulin powder. In the milk intervention period, the participants were given a minimum of 1 litre of Lærkevang whole-fat milk (Arla A/S) based on individual energy levels.

### Outcome measures

The primary outcome was changes in plasma ANGPTL4 concentration. The secondary outcomes were changes in blood lipid profile, characterisation of changes in faecal microbiota composition including changes in faecal SCFA content, changes in resting energy expenditure and lipid oxidation, changes in lipid and glucose metabolism, and *ANGPTL4* mRNA expression as well as gene expression of proteins related to obesity and hypoxia in adipose tissue. The secondary/exploratory outcomes also included a test of the effect of faecal water on ANGPTL4 expression in a human intestinal cell line. However the cells were not viable at relevant physiological concentrations and the assay was abandoned.

The participants were required to arrive at the department after a 12 h fast and to abstain from alcohol and strenuous physical activity for 48 h prior to the investigation days. The participants were asked to drink 500 ml of water during the fasting period. Additionally, they were asked to arrive via a transportation mode that did not require physical exertion. Upon arrival at the department, the participants were asked to void their bladder and were weighed. Their medical history and changes since their last visit were recorded.

Respiratory exchange was measured in a ventilated hood system (Jaeger Oxycon Pro Ventilated Hood system; Intramedic A/S) following 30 min of rest in supine position. Measurements were recorded over 25 min and were repeated once. The system was calibrated in the morning, prior to the measurements and between measurements. The accuracy of the ventilated hood was validated by an alcohol combustion test on a weekly basis with a CV of 1·5 %.

Body fat percentage was estimated with a hand-held bioimpedance device at screening (Tanita A/S). Participants were asked to remove all metals on their upper body and to stand erect, holding the device at shoulder height. Data on age, sex, height and weight were entered into the device prior to measurements. Dual-energy X-ray absorptiometry scans (Lunar Prodigy, GE Healthcare Danmark A/S) were performed once on the first day of the first dietary period. The participants were asked to remove all metal from their body and were scanned in the supine position in their underwear. They were instructed to lie still while being scanned. Weight was recorded in the fasted state, after voiding of the bladder, on a bathroom scale (Lindeltronic 8000S; Lindells), with the participant in their underwear. The participants were asked to distribute their weight evenly on both feet. Weight was recorded with an accuracy of 0·1 kg. Height was recorded on a wall-mounted stadiometer. The participant was asked to remove their shoes, stand erect with their heels together, arms to the side, and looking straight ahead. The participant was asked to take a deep breath and the measurement was performed before exhaling. Height was recorded with an accuracy of 0·5 cm.

Blood pressure and pulse were measured with an automated sphygmomanometer after a minimum of 15 min rest in the supine position. The measurement was repeated. To ensure accuracy of the measurements, blood pressure was assessed again if the difference between measurements exceeded 5 mm Hg for either systolic or diastolic blood pressure.

Faecal samples were collected for five consecutive days at the end of each intervention period. The participants were asked to collect all faecal matter in the period and to either store the samples on ice at home or deliver them to the department following collection. A small aliquot of the faecal samples was immediately frozen at −80°C for later analysis of the microbiota composition. Subsequently, all remaining samples were frozen until further treatment. The samples were then thawed at 5°C overnight. MilliQ water was added 1:1 (w/w) and the samples were homogenised with a rod blender and pooled with samples within participant and period. The pooled samples were frozen immediately at −80°C until analysis of NEFA.

Urine samples were collected over 24 consecutive hours at the start and end of each intervention period. The participants started urine collection in the morning on day 1 of the collection after voiding of the bladder. Collection was completed with the first voiding of the bladder on day 2 of collection. The participants were asked to take three *para*-aminobenzoic acid tablets of 80 mg during the collection period to monitor compliance. At the department, the urine samples were pooled, aliquoted and frozen at −80°C until analysis.

Fasting blood samples were collected at the first and last investigation day in all three periods. Additionally, a postprandial sample was collected 24 h after commencement of the intervention period. The samples for glucose analysis were collected in fluoride-coated vials. Samples designated for analysis of the lipid profile, C-reactive protein and insulin were collected in dry vials and allowed to stand for 20 min until coagulation. Samples for ANGPTL4 analysis were collected in EDTA-coated vials. All samples were centrifuged at 2500 ***g*** for 10 min and transferred to storage at −80°C until analysis.

An adipose tissue biopsy from the subcutaneous abdomen was performed upon completion of each intervention period. The biopsies were voluntary. Adipose tissue biopsies were performed according to standard operating procedure by trained medical staff. The area of skin was anaesthetised prior to injection of the biopsy needle (Pelomi Medical). The adipose sample was washed with sterile saline and blood and connective tissue was removed. Following cleaning, the sample was frozen immediately at −80°C in liquid N_2_.

### Sample size and randomisation

The sample size determination was based on changes in plasma ANGPTL4 concentration reported in a previous study^(^^6^^)^. The minimal difference in ANGPTL4 concentration was reported to be 3·3 ng/ml and the duration of the intervention period was 3 d. During this 21-d intervention, a minimal difference of at least 5 ng/ml was deemed realistic. Including twenty participants (eighteen completers) would provide 80 % power to detect this difference, allowing for a 10 % dropout at a 0·05 significance level. During the intervention, three participants dropped out and therefore an additional participant was recruited for the study (in accordance with the protocol).

The participants were randomly allocated to the sequence of the diet periods. The allocation sequence was computer-generated using randomly permuted blocks. The allocation of sequence was performed by a person that did not perform any measurements or tasks in the study, whereas another person produced the diets. A third person was responsible for including participants and for data analysis, and was blinded to the diet period sequence as well as the coding of the inulin and control diet. The allocation sequence was generated before commencement of enrolment and was not changed throughout the study. The participants were blinded to the inulin and control diet as they were visually identical, but due to the nature of the milk intervention, the participants and study staff were not blinded to this intervention. All study personnel that performed measurements were blinded to the inulin intervention throughout the study. Data were validated with double entries, performed individually by two members of the study staff. Statistical analysis was performed for all combinations of codes and documented before the codes were broken.

### Analyses

Glucose concentration at screening was measured using a single drop of blood from the finger tip of the participant and assessed with the Hemocue Glucose 201^+^ System (HemoCue AB). The participants were not required to be fasting and therefore inclusion was based on either a fasting blood glucose concentration of <7·0 mmol/l or a postprandial blood glucose concentration of <11·0 mmol/l.

The procedure for ANGPTL4 analysis has been described elsewhere^(^^11^^)^ and was performed with minor modifications. Briefly, plates were coated with anti-human ANGPTL4 antibody (RnD Systems, catalogue no. AF3485) diluted in filter-sterile PBS to reach a concentration of 0·2 mg/ml. The plates were then incubated at 5°C overnight on a rotating platform. The following morning, the plate was washed four times with washing buffer (PBS with a pH of 7·2–7·4, milliQ water and Tween^®^20) and incubated for 1 h with 300 µl of blocking solution in each well (10 % bovine serum albumin (BSA) in milliQ water). Samples were diluted 50-fold in diluent (RnD Systems, catalogue no. DY995). Recombinant human ANGPTL4 protein (RnD Systems, catalogue no. 3485-AN) was likewise diluted in diluent to produce a standard curve of 0, 0·3, 0·6, 0·9, 1·2, 1·5, 1·8 and 2·1 ng/ml. After four washes, the plates were incubated with 100 µl of sample per well and standard curve solutions for 2 h on a rotating platform before being washed four times and incubated with 100 µl of detection antibody solution (50 µg/ml solution of diluent and biotinylated anti-human ANGPTL4 antibody, RnD Systems catalogue no. BAF3485) for an additional 2 h. Subsequently, the plates were washed four times and incubated with a streptavidin horseradish peroxidase solution (PBS, 10 % BSA solution and streptavidin horseradish peroxidase) for 20 min on a rotating platform. Substrate (tetramethylbenzidine) was added after four washes and the plates were incubated for 8 min and subsequently the reaction was stopped with 50 µl of H_2_SO_4_. Optical density was then determined at 450 nm with a microplate reader (Spectra Rainbow; SLT Labinstruments).

Analysis of the microbiota composition was performed on a total of fifty-six faecal samples. Microbial DNA was extracted using a NucleoSpin soil kit (Macherey-Nagel) according to the manufacturer's instructions. PCR-based library formation, sequencing (Illumina MiSeq) and taxonomy assignment were performed as described in Holm *et al.*^(^^19^^)^. The microbiota composition was tested for differences due to diets or changes in plasma ANGPTL4 concentration from before and after intervention. Data analysis was performed in R v3.1.2 using the metagenomeSeq^(^^20^^)^, PhyloSeq^(^^21^^)^, Vegan: Community Ecology Package^(^^22^^)^ and GGplot2^(^^23^^)^ packages. The analysis was performed using filtered and normalised data, except for α diversity, which was calculated using raw, unfiltered data. The data were filtered by removing operational taxonomic units represented in fewer than three of the fifty-six samples or with a relative abundance across all samples smaller than 0·005 %. Read counts were normalised with metagenomeSeq using a cumulative-sum scaling in which raw counts were divided by the cumulative sum of counts up to a particular quantile. On average the dataset contained 32237 and 27602 sequences per sample before and after filtering, respectively.

Plasma samples from the beginning of each intervention period, 24 h after commencement of the intervention periods and after the periods were analysed for total NEFA concentration with the PENTRA 400 (HORIBA) (intra-serial CV 1·7 %). CV for glucose, cholesterol, HDL-cholesterol, LDL-cholesterol, TAG and insulin was 1·1, 0·4, 1·8, 1·4, 4·0, 3·9 and 7·1 %, respectively. Faecal homogenates, and plasma and urine samples were analysed for quantification of individual fatty acids at the end of each intervention. Data from faecal samples were adjusted for the total weight of the faecal collection.

Individual NEFA were quantified in biofluids (1 ml of urine and 1·8 ml of plasma) using the ethyl chloroformate free fatty acid (ECF-FFA) method^(^^24^^)^ and in faeces (0·1 g) with the modification from Amer *et al*^(^^25^^)^. ^2^H-labelled internal standards were added to the samples: 3 : 0, 6 : 0, and 16 : 0 to a final concentration of 0·3 mg/ml; C4 to 0·6 mg/ml; 5 : 0, 6 : 0, 8 : 0, 10 : 0, 12 : 0, 14 : 0 and 15 : 0 to 0·03 mg/ml; 17 : 0 to 0·15 mg/ml and 18 : 0 to 0·1 mg/ml. 15 : 0 concentration was too low to quantify. ECF, C_5_H_5_N, 18 : 2*n*-6, and 18 : 3*n*-3 standards were obtained from Sigma-Aldrich Chemie GmbH; anhydrous C_2_H_6_O, 3 : 0, 4 : 0, isobutyric acid, isovaleric acid, 2-methyl butyric acid, 5 : 0, 7 : 0, 9 : 0 and 11 : 0 standards, 3 : 0 (D5, 98 %), and 6 : 0 (D11, 98 %) internal standards were obtained from Sigma-Aldrich Chemie GmbH. 6 : 0, 8 : 0, 10 : 0, 12 : 0, 13 : 0, 14 : 0, 15 : 0, 16 : 0, 18 : 0 and 18 : 1*n*-9 standards were obtained from Fluka Chemie GmbH. C4 (D7, 98 %), 8 : 0 (D15, 98 %), 10 : 0 (D19, 98 %), 12 : 0 (D23, 98 %), 14 : 0 (D27, 98 %), 16 : 0 (D31, 98 %) and 18 : 0 (D35, 98 %) internal standards were obtained from Cambridge Isotope Laboratories, Inc., and the 15 : 0 (D29, 99·2 %) internal standard from CDN isotopes. Chloroform was obtained from Chem Solute, and NaOH from J. T. Baker. Ultrapure water came from a Milli-Q system (Millipore SAS), and anhydrous Na_2_SO_4_ was purchased from Merck.

NMR-based metabolomics was performed on a total number of 117 urine samples. ^1^H NMR spectroscopy was performed at 298 K on a Bruker Avance III 600 spectrometer, operating at a ^1^H frequency of 600·13 MHz, and equipped with a 5-mm ^1^H TXI probe (Bruker BioSpin). Standard one-dimensional spectra were acquired using a single 90° pulse experiment with a relaxation delay of 5 s. Water suppression was achieved by irradiating the water peak during the relaxation delay, and a total of sixty-four scans were collected into 32 K data points spanning a spectral width of 12·15 parts per million (ppm). Urine samples were thawed at room temperature, vortexed for 30 s, and centrifuged at 10 000 ***g*** for 5 min, 500 µl of supernatant were transferred to a 5 mm NMR tube and mixed with 100 µl of 0·75 m-PBS containing 0·5 % sodium trimethylsilyl propionate-d4 (TSP) in ^2^H-labelled water. Prior to Fourier transformation the data were multiplied by a 0·3 Hz line-broadening function. The proton NMR spectra were phase and baseline corrected manually using Topspin 3.2 (Bruker Biospin). All ^1^H spectra were initially referenced to the TSP signal at 0 ppm. After acquisition NMR spectra were profiled using Chenomx NMR Suite 8.1 (Chenomx). Ten metabolites were integrated and quantified using the Profiler module in the Chenomx NMR Suite. Each metabolite was manually checked in all samples in order to ensure a good fitting. As urine was collected over a 24-h period, the concentration of each metabolite is represented in units of mm/mm creatinine per 24 h.

The RNA was extracted from the adipose tissue samples using the RNeasy Lipid Tissue Mini kit (Qiagen Nordic). The adipose tissue samples were disrupted and homogenised with the use of the GentleMACS^™^ dissociator (Miltenyi Biotec Norden AB). The frozen samples together with lysis buffer (QIAzol lysis reagent) were added to the GentleMACS^™^ M tubes and the homogenisation was done according to the manufacturer's instructions. After a short incubation at room temperature, the samples were centrifuged. Subsequently, the RNeasy Lipid Tissue Mini kit protocol was followed. Centrifugation time was modified and increased to 30 min at step 5, 30 s at step 7, 9 and 10 and 2·5 min at step 11 in the manufacturer's protocol. Successful tissue homogenisation and extraction were ensured with the measurement of RNA quality and concentration (Nanodrop 1000 spectrophotometer; Thermo Fischer Scientific). cDNA synthesis was performed according to protocol with the use of TaqMan^®^ Reverse Transcription Reagents from Invitrogen^™^ (Life Technologies Europe BV). A concentration of 0·4 µg was used for a total of 20 µl RT reaction mix. The reverse transcriptase reaction mix was added to a ninety-six-well reaction plate together with the RNA solution for each sample and sealed with MicroAmp^®^ Optical Caps. The plate was placed in the Applied Biosystems RT PCR 7900HT system. The plate was heated to 25°C for 10 min, incubated at 48°C for 30 min and finally stopped by heating at 95°C for 5 min. Quantitative PCR was carried out with the use of TaqMan^®^ Gene Expression Assays for *ANGPTL4* (assay no.: Hs01101127_m1), hypoxia-inducible factor 1α (*HIF-1a*) (assay no.: Hs00153153_m1), glyceraldehyde-3-phosphate dehydrogenase (*GADPH*) (assay no.: Hs02758991_g1) and *LEP* (assay no.: Hs00174877_m1) (Life Technologies Europe BV), according to protocol. The final reaction mix was loaded to a ninety-six-well plate. Four replicates were made for each sample and a fast run was done with the Applied Biosystems RT PCR 7900HT system (Applied Biosystems International Inc.). *GAPDH* was included as a reference gene.

### Statistical methods

Statistical analyses were performed using R version 3.1.2 (The R Foundation for Statistical Computing, 2014). All dependent variables were controlled for normal distribution by histograms and the Shapiro–Wilk test for normality. Variables that were not normally distributed were transformed. ANGPTL4 plasma concentrations were not normally distributed and were log-transformed for analyses of associations. Differences between treatments were analysed in a mixed-effect model with participant as a random-effects factor. The models were adjusted for weight and age, and were validated with residual plots and normal quantile plots. The active treatments (the high-inulin and high-dairy diets) were compared with the control diet individually. The mRNA of the adipose tissue was analysed with the use of the comparative *C*_T_ method. Amplification plots were evaluated using the program ExpressionSuite Software version 1.0.3, the *C*_T_ value was identified and the relative quantification calculated. To determine if the expression of the examined genes was interdependent, the correlation coefficient was calculated between the Δ*C*_T_ values by Pearson's product moment correlation coefficient. To test for statistically significant differences in the microbiota composition, the Adonis test based on Bray–Curtis distance was used and metagenomeSeq for specific operational taxonomic units and phylotypes. Statistical significance was set to *P* < 0·05.

## Results

A total of twenty-one participants were included in the study (Table 1). Two participants withdrew during the study due to time constraints, and one was excluded due to lack of compliance. Eighteen participants completed the three intervention periods (nineteen participants completed the control period and were included in the analyses and twenty participants completed the high-dairy diet period and the high-inulin diet period).

**Table 1.** Baseline characteristics for all participants (Mean values with their standard errors for all included participants; *n* 21)

Parameter	Mean	se
Age (years)	32·9	0·85
Body weight (kg)	97·2	1·40
BMI (kg/m^2^)	29·3	0·5
Systolic blood pressure (mmHg)	122·1	1·5
Diastolic blood pressure (mmHg)	74·0	1·0
Pulse (beats per min)	55·0	0·8
Blood glucose (mmol/l)	5·6	0·1
Insulin (pmol/l)	70·5	6·0
TAG (mmol/l)	1·27	0·09
Total cholesterol (mmol/l)	4·54	0·07
LDL-cholesterol (mmol/l)	3·2	0·1
HDL-cholesterol (mmol/l)	1·17	0·02

ANGPTL4 concentration, blood lipid profile, insulin or glucose concentration did not change during the high-fat milk or the inulin intervention period, compared with the control period, nor did the diets have any effect on resting energy expenditure and lipid oxidation (Table 2). ANGPTL4 concentrations at the end of the intervention periods were not associated with lipid oxidation, TAG concentration or lipid profile (Table 2). Resting energy expenditure and lipid oxidation were not associated with ANGPTL4 concentration after adjusting for weight (Table 2). Total plasma NEFA concentration was not associated with plasma ANGPTL4 concentration at baseline or at the end of the intervention periods (Table 2). ANGPTL4 concentration was negatively associated with weight (*β* −0·016 (se 0·005), *P* = 0·001).

**Table 2.** Study end points after the intervention periods* (Mean values with their standard errors, and regression coefficients (*β*) with their standard errors)

	Control		Inulin diet		Inulin *v*. control			Milk diet		Milk *v*. control		
End points	Mean	se	Mean	se	*β*	se	*P*	Mean	se	*β*	se	*P*
ANGPTL4 (ng/ml)	40·2	4·2	39·7	2·4	0·04	3·40	0·93	37·8	2·50	−1·88	3·42	1·00
Glucose (mmol/l)	5·6	0·2	5·6	0·1	−0·017	0·17	1·00	5·6	0·1	0·024	0·17	0·99
Insulin (pmol/l)	69·1	9·7	67·5	7·6	−3·85	5·60	0·93	65·9	7·9	−6·22	5·67	0·72
TAG (mmol/l)	0·96	0·07	1·27	0·23	0·062	0·08	0·68	1·32	0·28	0·100	0·08	0·65
HDL-C (mmol/l)	1·1	0·04	1·1	0·04	−0·003	0·02	1·00	1·1	0·04	0·03	0·02	0·59
LDL-C (mmol/l)	3·1	0·13	3·2	0·10	0·12	0·07	0·30	3·1	0·13	0·07	0·07	0·80
Total cholesterol (mmol/l)	4·3	0·1	4·4	0·1	0·12	0·09	0·50	4·4	0·13	0·11	0·09	0·61
Energy expenditure (kcal/d)†	1737	40	1747	43·0	8·3	28·6	0·99	1754	31·9	2·1	28·6	1·00
Lipid oxidation (kcal/d)†	806	63	773	62·4	−38	62·8	0·96	888	65·8	74·2	62·8	0·66
Total NEFA (μmol/l)	411	46	389	30·7	−23	43·6	0·97	403	24·0	−7·3	43·8	1·00

ANGPTL4, angiopoietin-like protein 4; HDL-C, HDL-cholesterol; LDL-C, LDL-cholesterol.

* Data were analysed using mixed models with participants as the random effect. Estimates for transformed variables are reported from non-transformed models. All models were adjusted for age and weight. Significance was determined as *P* < 0·05.

† To obtain values in kJ/d, multiply by 4·184.

**Fig. 1.**
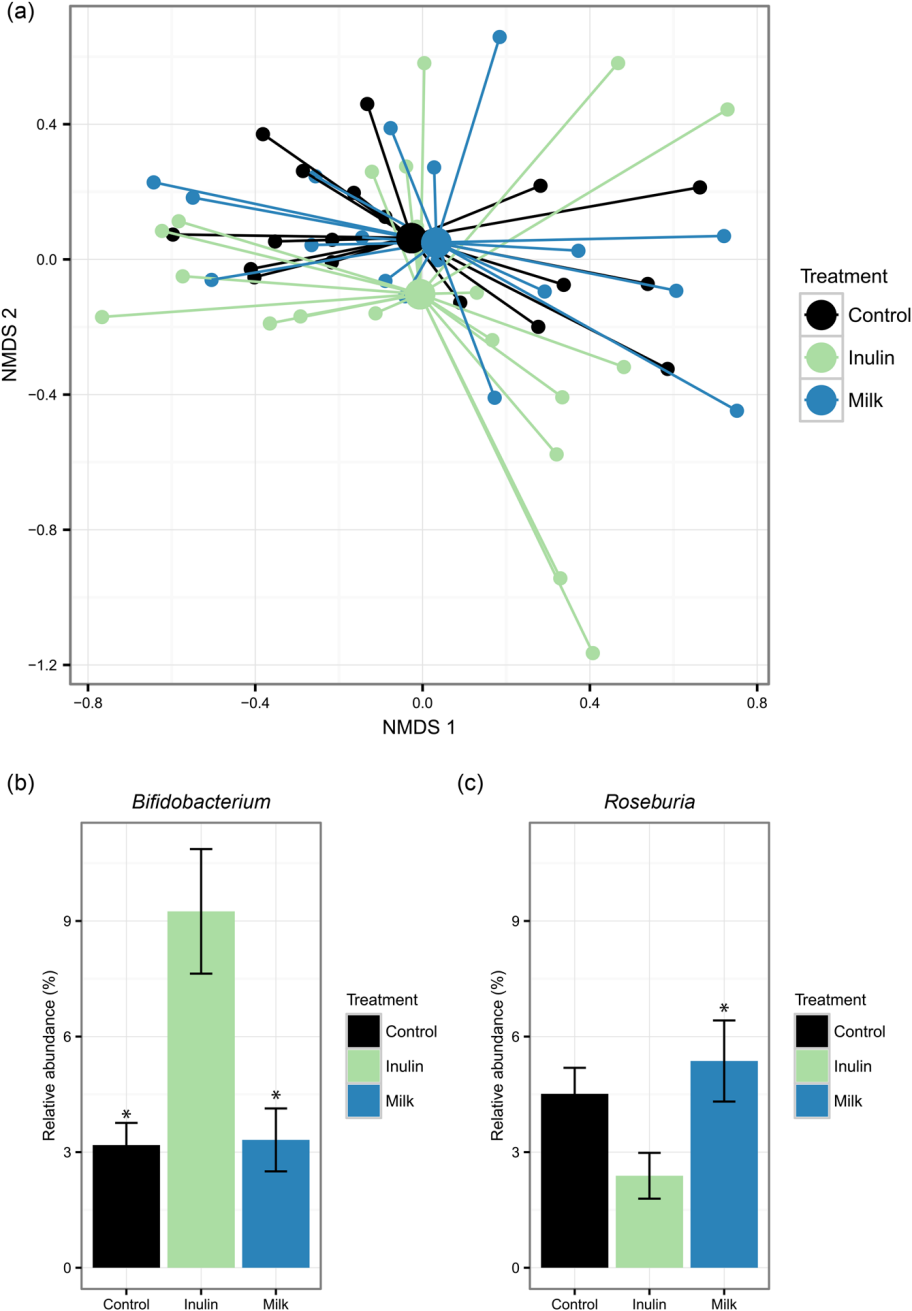
Diet-induced changes in the microbiota composition. (a) Non-metric multidimensional scaling (NMDS) plot using Bray–Curtis dissimilarity indices with centroids based on the treatments. Relative abundance of the genera found significantly affected by the diet: *Bifidobacterium* (b) and *Roseburia* (c). Values are means, with standard errors represented by vertical bars. * Statistically different relative abundance (adjusted *P* < 0·05) compared with the inulin treatment group.

The overall faecal microbiota composition did not change significantly in response either to milk or to inulin, compared with the control period (Fig. 1(a)). However, as the only detectable differences, we observed an increase in the relative abundance of bifidobacteria on the inulin diet, compared with the control diet, while the relative abundance of *Roseburia* was increased for the milk diet, compared with the inulin diet but not compared with the control diet (Fig. 1(c)).

There were no associations with either individual NEFA, total long-chain fatty acids or total SCFA and ANGPTL4 plasma concentration. Plasma concentrations of 16 : 0, 17 : 0, 18 : 2*n*-6 and 18 : 0 were significantly higher after the milk diet, compared with the control diet, in a model adjusted for weight and age (Table 3). Faecal concentrations of 12 : 0 and 16 : 0 were higher for the milk diet, compared with the control diet, whereas the concentration of 2-methyl-butyric acid was lower after the inulin period, compared with the control period (Supplementary Tables S1 and S2). C4 excretion in urine was not changed by either intervention diet, compared with control (Table 4). C4 excretion in urine was not associated with ANGPTL4 plasma concentration. Urine concentrations of 2-hydroxyisobutyrate, citrate and HCO_2_^–^ metabolites were higher after the milk diet compared with the control diet, whereas inulin did not change the concentration of urinary metabolites tested (Table 5). None of the additional urine metabolites tested was associated with plasma ANGPTL4.

**Table 3.** Plasma NEFA (μg/ml) after intervention periods* (Mean values with their standard errors)

	Control		Inulin		Milk			
Fatty acid	Mean	se	Mean	se	Mean	se	Inulin *v*. control: *P*	Milk *v*. control: *P*
12 : 0	1·1	0·4	0·7	0·06	0·76	0·10	0·27	0·45
14 : 0	1·8	0·2	1·7	0·2	2·17	0·15	1·00	0·52
16 : 1*n*-7	4·1	0·6	4·2	0·5	4·48	0·50	1·00	0·93
16 : 0	27·0	2·5	30·0	2·3	36·3	3·0	0·91	0·046
17 : 1	0·7	0·05	0·7	0·03	0·7	0·03	1·00	0·95
17 : 0	0·6	0·03	0·6	0·03	0·7	0·05	0·99	0·07
18 : 2*n*-6	16·5	1·6	19·2	1·5	23·7	2·4	0·80	0·02
18 : 1*n*-9	45·0	6·0	48·1	3·7	53·0	3·3	0·99	0·55
18 : 3*n*-3	1·6	0·2	1·4	0·2	1·9	0·2	0·95	0·57
18 : 0	13·5	0·8	14·7	1·0	17·9	1·6	0·95	0·02
∑ NEFA	111·1	11·5	120·7	8·6	141·3	9·7	0·95	0·12

* Data were analysed using mixed models with participants as the random effect. All models were adjusted for age and weight. Significance was determined as *P* < 0·05.

**Table 4.** Urine metabolites after intervention periods* (Mean values with their standard errors)

	Control		Inulin		Milk			
Metabolite	Mean	se	Mean	se	Mean	se	Inulin *v*. control: *P*	Milk *v*. control: *P*
2-Furoylglycine	0·007	0·002	0·006	0·002	0·01	0·003	0·85	0·61
2-Hydroxyisobutyrate	0·004	0·0002	0·004	0·0002	0·005	0·0002	0·10	<0·001
3-Hydroxyisovalerate	0·004	0·0004	0·004	0·0002	0·004	0·0003	0·92	0·87
Alanine	0·02	0·002	0·02	0·002	0·02	0·002	0·99	0·74
Citrate	0·1	0·03	0·2	0·03	0·2	0·04	0·19	<0·001
Dimethylamine	0·03	0·001	0·03	0·0008	0·03	0·001	0·85	0·46
Formate	0·02	0·002	0·02	0·002	0·03	0·003	0·11	0·01
Hippurate	0·2	0·04	0·2	0·04	0·2	0·04	0·99	0·99
Trigonelline	0·03	0·01	0·03	0·01	0·04	0·01	0·98	0·57
Trimethylamine *N*-oxide	0·01	0·002	0·02	0·01	0·01	0·003	0·53	1·00
Butyric acid (μg/ml)	22·7	3·2	24·4	3·1	21·3	3·4	0·99	0·99

* Data were analysed using mixed models with participants as the random effect. All metabolites except for butyric acid were normalised to 1 mm-creatinine. All models were adjusted for weight and age. Significance was determined as *P* < 0·05.

**Fig. 2.**
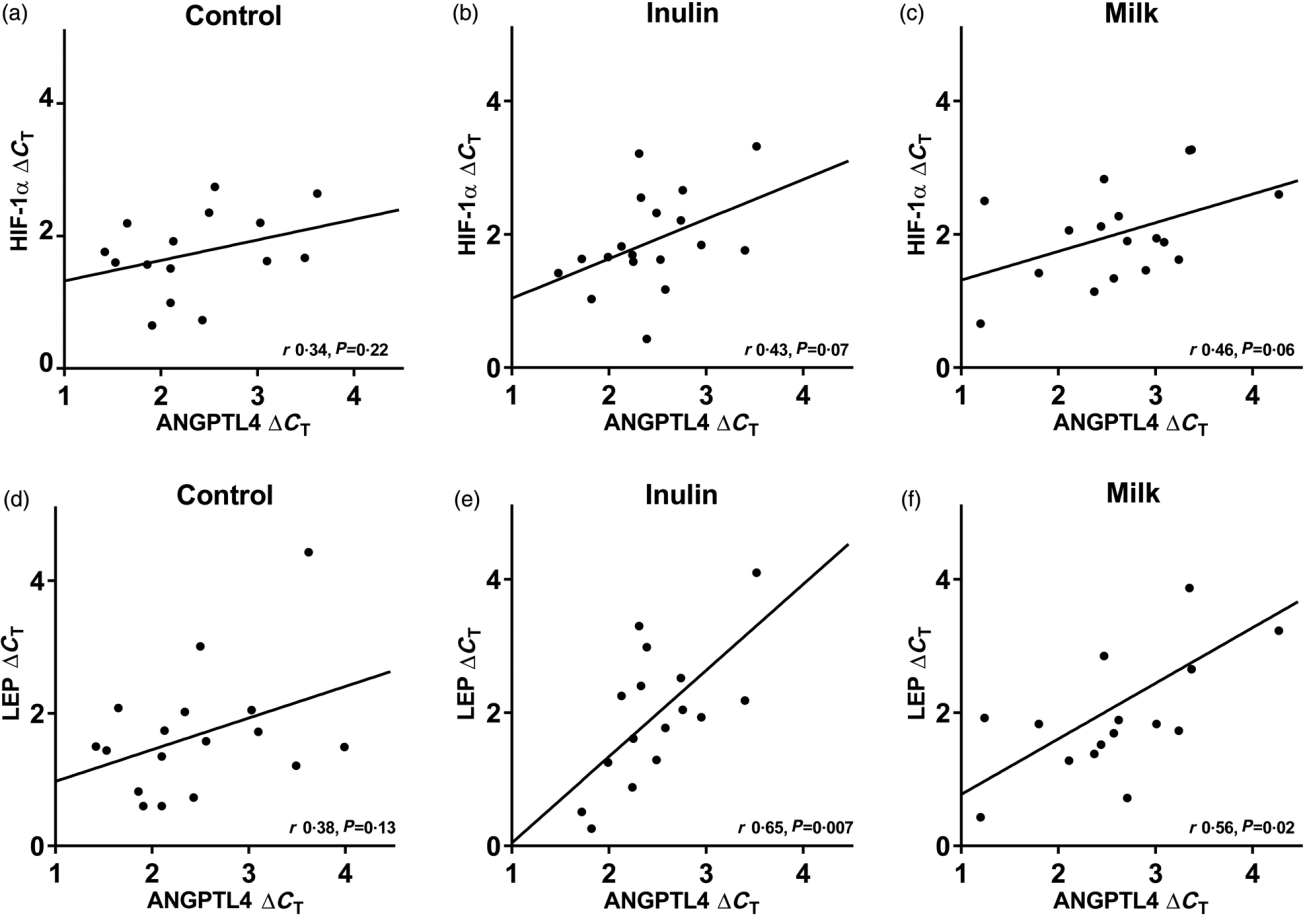
Correlations (*r*) between angiopoietin-like protein 4 (ANGPTL4) and hypoxia-inducible factor 1α (HIF-1α) mRNA in adipose tissue after control (a, *n* 15), inulin (b, *n* 18) and milk (c, *n* 17) diets, together with correlations between ANGPTL4 and leptin (LEP) mRNA after control (d, *n* 17), inulin (e, *n* 16) and milk (f, *n* 16) diets. Expressed in Δ*C*_T_ and calculated by Pearson's product moment correlation coefficient.

The *ANGPTL4* expression in adipose tissue was not affected by the diets and the correlation between *HIF-1α* and *ANGPTL4* mRNA did not reach significance for either diet (Fig. 2(a)–(c)). However, positive correlations between *ANGPTL4* and *LEP* mRNAs were observed in adipose tissue after the milk and inulin diets (Fig. 2(d)–(f)). Adipose tissue *ANGPTL4* mRNA was not associated with NEFA concentration, resting energy expenditure, lipid oxidation or TAG concentration (Table 5).

**Table 5.** Association between angiopoietin-like protein 4 (ANGPTL4) expression in adipose tissue and metabolism* (Regression coefficients (*β*) with their standard errors)

End points	*β*	se	*P*
TAG (mmol/l)	−0·25	0·40	0·90
NEFA (μmol/l)	−0·0008	0·0008	0·69
Resting energy expenditure	0·001	0·001	0·58
Lipid oxidation	−0·0004	0·0005	0·83

* Data were analysed using mixed models with participants as the random effect. Estimates for transformed variables are reported from non-transformed models. All models were adjusted for age and weight. Significance was determined as *P* < 0·05.

## Discussion

A 21-d intervention with either fibre or milk fat added to an isoenergetic standardised diet did not change ANGPTL4 plasma concentrations. Microbiota analyses showed only minor effects of the diets where inulin increased the relative abundance of bifidobacteria, compared with control, and milk increased the relative abundance of *Roseburia*, compared with inulin. Regulation of *ANGPTL4* expression through components of the gut microbiota was initially proposed by Bäckhed *et al*.^(^^14^^)^, who showed that expression of *ANGPTL4* was suppressed in the endothelium by conventionalisation of germ-free mice. Recently, Alex *et al*.^(^^26^^)^ showed that ANGPTL4 is produced by entero-endocrine cells, and Mattijssen *et al*.^(^^27^^)^ showed that ANGPTL4 inhibits pancreatic lipase. These findings indicate that ANGPTL4 regulates fat absorption in the gut in addition to regulation of lipid metabolism and storage by inhibition of lipoprotein lipase. Transwell studies have shown that ANGPTL4 is secreted to both the apical and basolateral side of enterocytes, indicating that there is a functional effect of luminal ANGPTL4^(^^28^^)^. The gut microbiota has been implicated in the development of obesity and several mechanisms have been proposed, including increased energy harvest^(^^16^^)^. Given the inhibitory properties of ANGPTL4 on pancreatic lipase, ANGPTL4 could provide a link between alterations in the microbiota and increased energy harvest. In this study, changes in the microbiota composition were not associated with ANGPTL4 plasma concentration. The plasma concentration of ANGPTL4 most probably represents other sources of ANGPTL4 in addition to the gut, such as the liver or adipose tissue. We have measured plasma ANGPTL4 and adipocyte *ANGPTL4* expression but we cannot account for the production and secretion of ANGPTL4 from the liver or the gut, which indicates that the observed minor changes in the microbiota were not sufficient to induce detectable changes in ANGPTL4 plasma concentration by changing the expression of ANGPTL4 in either the intestinal cells or the liver. Further, recent data from Dijk *et al.* suggest that ANGPTL4 is able to inhibit lipoprotein lipase intracellularly in adipocytes^(^^29^^)^, which could explain the lack of effects on plasma ANGPTL4 concentration. However, analyses of adipose tissue showed that *ANGPTL4* mRNA in the adipose tissue was not altered by the dietary interventions, suggesting that the negative results of the study are a result of a lack of effect on ANGPTL4.

Adipose tissue *ANGPTL4* mRNA was positively correlated with *LEP* mRNA in this study; however, the correlation with *HIF-1α* mRNA did not reach significance. ANGPTL4 is up-regulated in response to pronounced hypoxia in animals^(^^30^^)^ and in adipocytes^(^^12^^,^^31^^)^, which could be associated with an up-regulating effect of ANGPTL4 on angiogenesis.

Plasma NEFA concentration was not associated with plasma ANGPTL4 concentration in this study. Previous studies have found a positive association between ANGPTL4 plasma concentration and NEFA concentration^(^^6^^,^^11^^)^, and the interaction between NEFA and ANGPTL4 as well as the ability to affect or respond to NEFA concentration may be one of the most important functions of ANGPTL4 in relation to lipid metabolism. The most likely explanation as to why we do not find an association between plasma ANGPTL4 and plasma NEFA is that the magnitude of the interventions was markedly smaller in the present study, compared with the studies showing associations between ANGPTL4 and NEFA^(^^6^^,^^12^^)^. We did observe that several individual fatty acids were higher in plasma and faeces after the milk diet compared with the control diet, which was expected due to the higher dietary content of these fatty acids in the milk diet. However, there was no association between ANGPTL4 plasma concentration and these fatty acids. 12 : 0 and 16 : 0 are components found in bovine milk and, accordingly, the concentration of these fatty acids was higher in faecal samples collected after the milk diet. 16 : 0 was also increased in plasma samples collected after the milk intervention, compared with the control period. Previously, 12 : 0 has been shown to up-regulate *ANGPTL4* in *in vitro* studies^(^^13^^)^. However, the increased concentration observed in the faeces in the present study was not associated with plasma ANGPTL4 concentration.

In the present study excretion of HCO_2_^–^ was found to be increased in the milk diet. Excretion of HCO_2_^−^ has been linked to increased energy intake^(^^32^^)^ or suggested to be a secondary metabolite resulting from low fibre intake^(^^33^^)^. Collectively, excreted metabolites following a high-inulin or high-milk-fat dietary intervention did not demonstrate specific regulation of fatty acids in this study, which is in accordance with the other results from the study.

The study was highly controlled by standardisation of the diets and the crossover design, which reduced inter-individual variation by allowing for participants to be compared with themselves. A previous study of ANGPTL4 has tested extreme physiological conditions, such as prolonged fasting or high amounts of fat^(^^6^^)^, whereas this study tested the effects of ANGPTL4 during a physiologically relevant nutritional intervention for a longer period. The magnitude of the interventions is a likely cause of the different results of the two studies.

In conclusion, this study does not support that ANGPTL4 can be effectively modified by moderate nutritional interventions by a modification of the gut microbiota composition.

## Supplementary material

For supplementary material accompanying this paper visit http://dx.doi.org/10.1017/jns.2016.38. click here to view supplementary material
