# Supplementary material for: A randomised, controlled, crossover study of the effect of diet on angiopoietin-like protein 4 (ANGPTL4) through modification of the gut microbiome
Source: J Nutr Sci. 2016 Dec 6;5:e45. doi: 10.1017/jns.2016.38 (PMC5465810; doi:10.1017/jns.2016.38)
Supplement: Supplementary file 1 [file S2048679016000380asup001.doc]

Supplementary Table S1. Long-chain fatty acids in faecal samples

| **Long chain fatty acid (mg/5 days)** | **Control** | | **Inulin** | | **Milk** | | **Inulin vs. Control p** | **Milk vs. Control**  **p** |
| --- | --- | --- | --- | --- | --- | --- | --- | --- |
|  | **Mean** | **SE** | **Mean** | **SE** | **Mean** | **SE** |  |  |
| C12:0 | 1.5 | 0.8 | 1.4 | 0.5 | 3.8 | 0.9 | 1.00 | 0.049* |
| C13:0 | 0.3 | 0.1 | 0.3 | 0.1 | 0.6 | 0.1 | 0.99 | 0.50 |
| C14:1 | 1.5 | 0.3 | 1.1 | 0.2 | 1.1 | 0.1 | 0.59 | 0.69 |
| C14:0 | 11.8 | 6.8 | 10.3 | 2.8 | 18.5 | 3.9 | 0.99 | 0.80 |
| C15:0 | 8.4 | 1.0 | 7.5 | 1.0 | 12.1 | 1.4 | 0.96 | 0.07 |
| C16:1*n*-9 | 2.4 | 0.4 | 1.8 | 0.2 | 19.4 | 16.0 | 1.00 | 0.60 |
| C16:0 | 255.4 | 25.1 | 272.4 | 34.7 | 388.5 | 38.9 | 0.99 | 0.03* |
| C17:1 | 7.5 | 4.6 | 2.7 | 0.3 | 2.5 | 0.2 | 0.56 | 0.54 |
| C17:0 | 13.9 | 6.0 | 7.0 | 0.9 | 11.1 | 1.2 | 0.51 | 0.96 |
| C18:2*n*-6 | 76.5 | 10.5 | 61.0 | 7.2 | 69.4 | 8.1 | 0.55 | 0.93 |
| C18:1*n*-9 | 183.4 | 20.7 | 146.2 | 17.1 | 192.7 | 22.6 | 0.49 | 0.99 |
| C18:3*n*-3 | 6.2 | 1.1 | 5.9 | 0.7 | 5.6 | 0.4 | 0.99 | 0.89 |
| C18:0 | 459.0 | 65.6 | 394.3 | 50.8 | 577.1 | 62.1 | 0.88 | 0.37 |
| Ʃ LCFA | 1328.1 | 212.8 | 907.4 | 110.2 | 1263.9 | 127.2 | 0.09 | 0.40 |

Data represent mean ± standard error (SE). Mean values are calculated based on total fecal mass collected over 5 days at the end of each intervention. Data was analyzed using mixed models with participants as random effect. All models were adjusted for age and weight. Significance was determined as p<0.05. LCFA, Long chain fatty acid.

Supplementary Table S2. Short-chain fatty acids in 5-day faecal samples

| **Short chain fatty acid (mg/5 days)** | **Control** | | **Inulin** | | **Milk** | | **Inulin vs. Control**  **p** | **Milk vs. Control**  **p** |
| --- | --- | --- | --- | --- | --- | --- | --- | --- |
|  | **Mean** | **SE** | **Mean** | **SE** | **Mean** | **SE** |  |  |
| Propionic acid | 138.1 | 16.1 | 152.6 | 25.1 | 114.5 | 12.7 | 0.98 | 0.50 |
| Isobutyric acid | 123.2 | 20.9 | 85.4 | 12.2 | 127.5 | 15.2 | 0.33 | 1.00 |
| Butyric acid | 656.4 | 73.6 | 788.9 | 123.9 | 616.0 | 93.0 | 0.82 | 0.95 |
| 2-methyl butyric acid | 9.9 | 1.6 | 6.2 | 0.7 | 8.1 | 0.9 | 0.03* | 0.66 |
| Isovaleric acid | 12.9 | 1.9 | 8.9 | 0.9 | 10.3 | 1.1 | 0.08 | 0.35 |
| Valeric acid | 21.8 | 2.7 | 22.4 | 4.3 | 17.7 | 1.9 | 1.00 | 0.33 |
| ƩSCFA | 962.2 | 104.1 | 1064.3 | 151.8 | 894.1 | 114 | 0.98 | 0.94 |

Data represent mean ± standard error (SE). Mean values are calculated based on total fecal mass collected over 5 days at the end of each intervention. Data was analyzed using mixed models with participants as random effect. All models were adjusted for age and weight. Significance was determined as p<0.05. SCFA, short chain fatty acid.
